# Effect of Cadmium on Organ Biomarkers and Evaluation of Certain Adaptogens in Broilers

**DOI:** 10.4103/0971-6580.75856

**Published:** 2011

**Authors:** G. Swapna, A. Gopala Reddy

**Affiliations:** Department of Pharmacology and Toxicology, College of Veterinary Science, Rajendranagar, Hyderabad - 500 030, India

**Keywords:** Cadmium, *Emblica officinalis*, polyherbal formulation, vitamin E

## Abstract

Day-old male broiler chicks were randomly divided into 8 groups consisting of 10 chicks in each. Groups 1 and 2 were maintained as plain control and cadmium (100 ppm in feed) toxic control, respectively, for 6 weeks. Groups 3, 4 and 5 were maintained on a combination of cadmium along with **Emblica* officinalis* (500 ppm in feed), vitamin E (300 ppm in feed) and polyherbal formulation (1 g/kg feed), respectively, for 6 weeks. Groups 6, 7 and 8 were maintained on cadmium for the first 4 weeks and on **E. officinalis**, vitamin E and polyherbal formulation, respectively, during the subsequent 2 weeks without cadmium. The biochemical parameters such as alanine transaminase (ALT), alkaline phosphatase (ALP), urea and creatinine were significantly (*P*<0.05) elevated in toxic control. These parameters revealed improvement following treatment with *E. officinalis*, vitamin E and polyherbal formulation in groups 6, 7 and 8, respectively. Thus, it is concluded that supplementation of *E. officinalis*, vitamin E and polyherbal formulation in feed is useful in preventing and treating the cadmium-induced toxic effects.

## INTRODUCTION

Cadmium is a hazardous heavy metal that is widely distributed in the environment, and it is present in trace levels in sea water and in a broad range of animal and plant species.[1] Many of the poultry industries are located near highways and in the proximity of industries that liberate Cd as pollutant. So, it is a common finding that poultry industry is affected with Cd toxicity. Cd is a potent inducer of apoptosis, which is mediated via induction of oxidative stress.[2] Cadmium is also known to cause stress by increasing lipid peroxidation or by changing intracellular glutathione (GSH) levels.[3] Keeping these toxic effects in consideration, an experimental study was conducted to evaluate its effects on certain organ biomarkers and to evaluate the benefit of using *Emblica officinalis*, vitamin E and polyherbal formulation.

## MATERIALS AND METHODS

A total of 80 day-old sexed male broiler chicks of *Cobb* strain obtained from Venkateswara Hatcheries (Hyderabad) were randomly divided into eight groups consisting of 10 chicks in each. Group 1 was maintained on basal diet for 6 wk, group 2 on cadmium (100 ppm in feed) for 6 wk, group 3 on a combination of Cd + **E. officinalis** (500 ppm in feed) for 6 wk, group 4 on a combination of Cd + vitamin E (300 ppm in feed) for 6 wk, group 5 on a combination of Cd + polyherbal formulation (1 g/kg feed) (stressroak; Ayurvet Ltd., Himachal Pradesh), consisting of *Withania somnifera, Ocimum sanctum*, Shilajit, Amla, *Mangifera indica*) for 6 wk. Groups 6, 7 and 8 were fed with Cd for the first 4 wk followed by E. *officinalis*, vitamin E and polyherbal formulation, respectively, during the subsequent 2 wk.

Sera samples were separated for the estimation of renal and hepatic biomarkers by using commercially available diagnostic kits (Qualigens Pvt. Ltd., Mumbai, India). The data were analyzed by one-way analysis of variance (ANOVA) using statistical package for social sciences (SPSS), version 10. *P*<0.05 was considered as significant.

## RESULTS AND DISCUSSION

The activity of alanine transaminase (ALT; IU/l) in group 1 was 19.346 ± 0.131, which was significantly (*P*<0.05) increased in Cd toxic control groups 2, 6, 7 and 8 to 60.279 ± 0.166, 60.115 ± 0.022, 59.728 ± 0.208 and 60.030 ± 0.080, respectively, at the end of 4^th^ wk. In groups, 6, 7 and 8 following treatment, the activity was significantly (*P*</0.05) reduced to 41.743 ± 0.331, 42.075 ± 0.056 and 40.023 ± 0.088, respectively, as compared to their respective 4^th^ wk values and that of group 2 (68.071 ± 0.012) at the end of 6^th^ wk.

The activity of alkaline phosphatase (ALP; units/ml) in group 1 was 70.785 ± 0.620, which was significantly (*P*<0.05) increased in the Cd toxic control groups 2, 6, 7 and 8 to 80.211 ± 1.149, 78.674 ± 0.515, 78.206 ± 0.337 and 78.161 ± 0.314, respectively, at the end of 4^th^ wk. In groups 6, 7 and 8 following treatment, the ALP activity was significantly (*P*<0.05) decreased (72.034 ± 0.065, 70.061 ± 0.198 and 70.04 ± 0.012, respectively) when compared to their respective 4^th^ wk values and that of group 2 (82.693 ±0.216) at the end of 6^th^ wk.

The activities of ALT and ALP are elevated following hepatocellular injury.[4] Demerdesh *et al*.[5] reported an increase in the activity of ALT and ALP in plasma, when rats were fed with Cd at 5 mg/kg. Treatment with *E. officinalis*, vitamin E and stressroak following discontinuation of cadmium resulted in a significant reduction in the activity of ALT. The hepatocellular injury due to cadmium could be attributed to the cadmium-induced generation of free radicals and the reversal of the findings following treatment could be attributed to the antioxidant and the hepatoprotective potential of the drugs in test. Alcoholic and aqueous extracts of the fruits of *Emblica* have shown hepatoprotective properties in experiments in rats; it inhibited hepatic lipid peroxidation and the increase of serum levels of ALT, aspartate transaminase (AST) and lactate dehydrogenase (LDH). These results support the use of *Emblica* fruit for hepatoprotection.[6] However, simultaneous supplementation of drugs in the test along with cadmium revealed a significant increase in ALT and ALP activities at different time intervals when compared to the plain basal diet control, but the values were significantly lower in comparison to pure cadmium toxic control group 2. This finding suggests the prophylactic potential of these drugs to prevent cadmium-induced toxic manifestations, though there was no complete prevention of changes.

The renal indices such as serum urea and creatinine were significantly (*P*<0.05) increased in toxic controls 2, 6, 7 and 8 at the end of 4^th^ wk when compared to group 1 [Table 1]. This could be due to the oxidative damage of cadmium on renal tissue. However, in groups 6, 7 and 8 following treatment with *E. officinalis*, vitamin E and polyherbal formulation, respectively, the renal profile revived to normal, at the end of 6^th^ wk, suggesting the involvement of oxidative damage prior to treatment. Non-protein nitrogenous (NPN) substances such as serum urea and creatinine are increased only when renal function is below 30% of its original capacity in birds. Plasma urea appears to be the single most useful variable for detection of pre-renal causes of renal failure.[4] In the present study, the serum urea and creatinine levels were significantly increased in toxic controls at the end of 4^th^ wk as compared to the remaining groups. The nephrotoxic metal cadmium at micromolar concentrations induces apoptosis of rat kidney proximal tubule (PT) cells within 3-6 hours of exposure. This involves a complex and sensitive interplay of signaling cascades involving mitochondrial pro-apoptotic factors, calpains and caspases, whose activation is determined by cadmium concentration and the duration of cadmium exposure.[7] Cadmium-induced apoptosis in rat kidney epithelial cells involves decrease in nuclear factor-kB (NF-kB) activity.[8] Treatment in groups 6, 7 and 8 with *E. officinalis*, vitamin E and stressroak, respectively, following discontinuation of cadmium, resulted in significant decrease in serum creatinine and blood urea nitrogen (BUN) as compared to Cd toxic control group 2, which suggests their protective role. However, simultaneous supplementation of drugs in test along with cadmium revealed a significant increase in serum creatinine and BUN at different time intervals when compared to basal diet control, though the values were significantly lower in comparison to pure cadmium toxic control group 2. This finding suggests the prophylactic potential of these drugs to prevent cadmium-induced toxic manifestations, though there was no complete prevention of the changes. The beneficial renal protective actions of drugs in test may be attributed to their antioxidant/free radical scavenging actions and protection of protein thiols from deleterious actions of cadmium in kidney.

In conclusion, the study revealed that cadmium has the potential to induce hepatotoxicity and nephrotoxicity and supplementation with **E. officinalis**, vitamin E and polyherbal formulation (stressroak) has a beneficial role in preventing the adverse effects.

**Table 1:** Profile of renal biomarkers

Group	Creatinine (mg/dl)		Urea (mg/dl)	
	4^th^ wk	6^th^ wk	4^th^ wk	6^th^ wk
Basal diet (1-42 days)	0.343±0.018^aA^	0.424±0.046^aB^	16.752±0.035^aA^	17.208±0.149^aA^
Cadmium (1-42 days)	2.854±0.021^cA^	3.235±0.012^eB^	43.589±0.194^dA^	49.287±0.245^eB^
Cadmium+ **Emblica* officinalis* E (1-42 days)	2.277±0.031^bA^	2.232±0.073^bA^	33.332±0.341^cA^	33.047±0.168^dA^
Cadmium + vitamin E (1-42 days)	2.239±0.312^bA^	2.216±0.011^bA^	32.193±0.245^cA^	30.199±0.168^cB^
Cadmium + stressroak (1-42 days)	2.276±0.211^bA^	2.214±0.011^bA^	26.210±0.266^bA^	25.925±0.381^bA^
Cadmium (1-28 days); **Emblica* officinalis* (29-42 days)	2.856±0.231^cA^	2.443±0.064^dB^	43.304±0.168^dA^	31.338±0.266^cB^
Cadmium (1-28 days); vitamin E (29-42 days)	2.823±0.181^cA^	2.365±0.031^cB^	43.019±0.133^dA^	31.345±0.206^cB^
Cadmium (1-28 days); stressroak (29-42 days)	2.876±0.041^cA^	2.395±0.051^cdB^	42.450±0.320^dA^	30.758±0.207^cB^

Values are mean + SE of 6 observations, Means with different alphabets as superscripts differ significantly (*P*<0.05) ANOVA. Capital alphabets (Horizontal comparison) Small Alphabets (Vertical comparison)
